# p130 And pRb in the Maintenance of Transient Quiescence of Mesenchymal Stem Cells

**DOI:** 10.1155/2020/8883436

**Published:** 2020-09-18

**Authors:** Boris Popov, Nikolai Petrov, Vladimir Ryabov, Igor Evsyukov

**Affiliations:** ^1^Institute of Cytology Russian Academy of Sciences, St. Petersburg 194064, Russia; ^2^Children's Hospital, The Ohio State University, Columbus, OH 43205-2696, USA

## Abstract

An effective regulation of quiescence plays a key role in the differentiation, plasticity, and prevention of stem cells from becoming malignant. The state of quiescence is being controlled by the pRb family proteins which show overlapping functions in cell cycle regulation; however, their roles in controlling the proliferation of mesenchymal stem cells (MSCs) remain to be understood. This study investigated the regulation of transient quiescence using growth curves, proliferation assay, the cytometric evaluation of cell cycle, Western blotting, and the electromobility gel shift assay (EMSA) on synchronized MSCs of the C3H10Т1/2 and control cells with different statuses of pRb proteins. It has been found that functional steady-state level of p130 but not pRb plays a critical role for entering, exiting, and maintenance of transient quiescence in multipotent mesenchymal stem cells.

## 1. Introduction

pRb, p130, and p107 comprising the pocket protein family are considered ubiquitous cell cycle regulators with overlapping functions [1]. All three pocket proteins are required to be inactivated for the loss of cell cycle control, ability to differentiate, and induction of cell immortality [2]. These observations support the idea of the functional redundancy of pocket proteins whose physiological relevance is currently not completely clear [3]. On the other hand, pocket proteins regulate the expressions of various cell cycle genes during distinct cell cycle phases [4]. Furthermore, pRb deficiency targets genes that encode cell cycle regulatory proteins, the expression of which is regulated by E2f1-3 [5, 6]. In contrast, the loss of p107/p130 alters the expression of genes regulating quiescence in response to growth or differentiation signals [6].

The orthologs of pocket proteins were found in some unicellular organisms but appear in almost all multicellular organisms [7] and are well conserved from plants to animals [8]. The corresponding ancestral genes were divided into *RB1* (including *RB1*) and *RBL* (including *RBL1/p107* and *RBL2/p130*) subgroups. The members of the *RBL* subgroup show more similarity in sequence with *RB* ancestral sequences than the members of the *RB1* subgroup and represent more ancient functions of pRb signaling found to be associated with the control of quiescence and cell fate choice; additionally, pRb can also contribute to the development of tumor suppression via controlling all the aspects of cell cycle and coupling it with the processes of differentiation, cell senescence, and apoptosis [9].

The major targets of pRb proteins include the E2f transcription factors that transform the pocket protein activity into the transcription of genes associated with cell cycle regulation. E2fs are divided into activators (E2f1-3) and suppressors (E2f4-5) of transcription. Е2f4-5 accumulate in quiescence and bind р107/р130 while Е2f1-3 are expressed in G1/S transition and exhibit high affinity for pRb. The distinction in the ability to bind different E2fs allows pocket proteins to regulate various E2f-responsive genes [4]. However, under pRb deficiency, p130/p107 may bind activating E2fs and change their functional activity [10].

Different types of stem cells decide whether to differentiate or not and select a tissue-specific cell fate during intrinsic cell division. Furthermore, self-renewing embryonic stem cells (ESCs) do not have the R1 check point due to the functional inactivation of pRb signaling. They do not produce p130 and express in G1 phase hyperphosphorylated and functionally inactive pRb. However, pRb signaling becomes functional in differentiated ESCs. Modern reports suggest that pocket proteins have emerged as important regulators of stem cell fate. This pRb protein function is highly conserved in evolution and associated with development, tissue maintenance, and regeneration [11, 12]. In contrast to ESCs, the adult stem cells stay in quiescence due to the active status of pRb proteins [13]. A current adult stem cell model suggests that in tissues with active proliferation, bone marrow, epidermis, and intestine, two populations of quiescent and cycling somatic stem cells coexist, which may replace each other in the course of native regeneration [14]. MSCs represent a highly heterogeneous population [15] which, similar to other tissue-specific stem cells, may include quiescent and actively proliferative interchangeable pools of stem cells. The regulation of MSCs' quiescence and the contribution of separate pocket proteins remain to be investigated.

This study aims at investigating the role of pRb and p130 in the maintenance of the quiescent state in MSCs compared to those in the somatic-differentiated cells with different functional status of pocket proteins. It has used the mouse embryonic polypotent fibroblasts of the C3H10T1/2 (10T1/2) cell line as MSCs. 10T1/2 is a multipotential cell line that can be converted by 5-azacytidine treatment or growth in special medium to myogenic, chondrogenic, adipogenic lineages, and exploited worldwide as a model to study mesodermal differentiation [16, 17]. The 10T1/2 cells are preferable for the study of cell cycle regulation in comparison with native adult MSCs because the doubling time of native MSCs from different tissues continuously increases in each cell cycle due to intrinsic replicative senescence [18]. To study the roles of pRb/p130 in the maintenance of the MSCs' quiescent state, this study has used the cells of different phenotypes. Cell cycle proteins are highly conserved and show functional activity when being transferred from the human to the yeast cells [19]. This allows exploiting the heterogeneous system in studying the functional roles of pocket proteins in MSCs. Pocket proteins are fully functioning in the established human T98G cell line which has been used as an appropriate source for study of the p130 role in cell cycle regulation [20].

First, this study evaluated the differential potential of 10T1/2 cells and induced them to adipogenic differentiation undergrowth in a special medium. Then, it compared the growth and proliferation rates of 10T1/2, T98G somatic differentiated cells expressing functional pocket proteins [20] and HeLa cells with virally inactivated pocket proteins [21] under normal and serum deprivation conditions using growth curves and the MTT test. Its second aim was to determine the cell cycle-associated alteration of p130 and pRb in synchronized MSCs and control cell lines by the technique of Western blotting. Its third goal was to determine the composition of pocket proteins-E2f-DNA complexes (pp-E2f-DNA) in synchronized cells of different phenotypes by EMSA. This study has shown that under serum-deprived conditions, MSCs, similar to T98G cells with functional pocket proteins, enter quiescence and accumulate in the G0/G1 phase in contrast to HeLa cells that continue to proliferate. In the quiescent state, MSCs and T98G cells form the p130/E2f4-DNA complex that disappears in proliferating cells in exchange for the formation of the pRb-E2f1-DNA complex. In contrast, HeLa cells do not produce p130 and do not form the p130-E2f4-DNA complex, however, they produce pRb and form the pRb-E2f1-DNA complex under serum-deprived and full-growth medium conditions. The results suggest that the entering and maintenance of the quiescent state in MSCs is associated with the functional steady-state level of p130 but not pRb.

## 2. Material and Methods

### 2.1. Cell Culture and Adipogenic Differentiation

10T1/2, T98G, and HeLa cell lines were obtained from the American Type Cell Collection. The 293HEK cells were gifted by Dr. Tomilin (Institute of Cytology, Sankt-Petersburg, Russia). The cells were grown in DMEM supplemented with 10% fetal calf serum (FCS). For the induction of adipogenic differentiation (AD), 10^4^ cells in the logarithmic growth phase were seeded on coverslips in 35 mm plates in 1.5 mL growth medium. The following day, the growth medium was replaced with a differentiation medium comprising 50 *μ*g/mL gentamicin, 10% FCS, 5 *μ*g/mL insulin, 50 *μ*M indometacin, 10–5 M dexamethasone, and 0.5 *μ*M 3-isobutyl-1-methylxanthine (Sigma, United States). The cells were fixed with 4% paraformaldehyde in PBS 1 h before the induction of AD and in 10 days after. The fixed cells were washed twice with distilled water and once with 60% isopropanol (Vekton, Russia), air dried, stained with Oil Red (Sigma, United States), and washed four times with distilled water. The working dye solution was prepared by mixing six parts of the solution containing 25 *μ*g/mL Oil Red in 100% isopropanol with four parts of distilled water. Before cell treatment, the solution was filtered through a 0.45 *μ*m filter. The cells were observed under the Pascal microscope (Carl Zeiss, Germany) with transmitted light using a 10× objective.

### 2.2. Growth Curves, Growth Synchronization, and Flow Cytometry

For cell cycle synchronization, the cell cultures of 90% density were grown in DMEM supplemented with 0.15% of FCS for 72 h (0 h point) followed by restimulation with 10% of FCS to obtain the probes every 6 hours. To estimate the growth curves, the exponentially growing cells were cultured in a 96-well plate at 2 × 10^4^ cells per well in triplicate in the full growth medium containing 10% FCS (FM) and the serum-starved medium containing 0.15% FCS (SFM). The number of living cells was detected using a hemocytometer and 0.4% trypan blue (Thermo Fisher Scientific, USA) to assess cell viability using the dye exclusion test. The dead cells were excluded from the calculations. The cells were counted on a daily basis until day 4 to estimate the mean and standard deviation for each cell line. For flow cytometry analysis, the synchronized cells were trypsinized and washed twice in PBS containing 1% bovine serum albumin. For nuclear staining, the cells were resuspended in an appropriate volume of PBS containing 250 *μ*g/ml of RNase A and 50 *μ*g/ml of propidium iodide to make the 5 × 10^5^ cell/ml concentration and then incubated at 37°C for 30 min. Flow cytometry analysis was performed using the cytometer ATC 1000 (Bruker, Germany) and analyzed as in [22].

### 2.3. Proliferation Rate

Cell proliferation rate was estimated using the Cell Proliferation Kit I MTT (Sigma-Aldrich, USA) based on the manufacturer's recommendation. The asynchronously growing 10T1/2, T98G, and HeLa cells were seeded at a concentration of 2 × 10^4^ cells/well in 100 *μ*l full culture medium in 24 wells of 96 well cell culture plate (PPT, Russia) and incubated for 24 h. The following day, the medium was removed and the cells were washed twice with 100 *μ*l of PBS. Thereafter, 12 wells containing cells of each cell line were filled with full growth medium (FM) while the other 12 were filled with serum-free medium (SFM). For the next four days, 10 *μ*l of the MTT labeling reagent was added on a daily basis to each well, and the cells were placed into a CO_2_ incubator for 4 h. Then, 100 *μ*l of the solubilization solution was added to each well and the plate incubated overnight in the CO_2_ incubator. The following day, the content of each plate was transferred to the corresponding ELISA plate (Jet Bio-filtration Co., China) followed by measuring OD by the ELISA reader at 570 nm.

### 2.4. Protein Electrophoresis and Immunoblotting

The proteins from the cell extracts were subjected to electrophoresis in 8% polyacrylamide gel with SDS. After electrophoresis, the proteins were transferred from the gel onto a PVDF membrane (Millipore, USA) and visualized with specific antibodies and a chemiluminescence reagent (ECL, Sigma, USA) as described earlier in [22].

### 2.5. Electrophoretic Mobility Gel Shift Assay (EMSA)

For EMSA, T98G, 10T1/2, HeLa, and 293HEK cells were grown on 100 mm plastic dishes and synchronized as described above. To prepare the nuclear extracts, the cells were lysed in a buffer containing 10 mM HEPES (pH 7.9), 0.1 mM EGTA (pH 8.0), 0.1 mM EDTA (pH 8.0), 10 mM KCl, and 0.5 mM PMSF. Additionally, NP-40 was added to the final concentration of 0.6%. The lysates were shaken on ice for 5 min and then centrifuged at 14000 rpm for 1 min. The pellets were resuspended on ice in the following buffer—20 mM HEPES (pH 7.9), 1 mM EDTA (pH 8.0), 1 mM EGTA (pH 8.0), 0.4 M NaCl, 1 mM DTT, 1 mM PMSF, and 1 mg/ml aprotinin, leupeptin, and pepstatin for 30 min, and then spun down at 14000 rpm for 20 min at +4°C. The protein concentration in the nuclear extracts was determined by the Bradford assay, and the extracts containing 20 *μ*g of the total protein were incubated in a volume of 10 *μ*l in the loading buffer—1 mg/ml of salmon sperm DNA in 10 mM HEPES (pH 7.9), 15% glycerol, 1 mM EDTA, 8 mM MgCl_2_, and 1 mM DTT for 15 min on ice. The oligonucleotides with 5′residues overhung for the E2F-binding sites of the Ad5E2 promoter (5′-CGTACTTTTCGCGCTTAAATTT-3′ and 5′-CAAATTTAAGCGCGAAAAGTAC-3′) were synthesized, annealed, and labeled using the Klenow fragment of *Escherichia coli* DNA polymerase I and [*α*-^32^P] dGTP. Competition (C), deoxycholate (D), and antibody treatment were carried out as described earlier [22]. The E2F-binding complexes were resolved by electrophoresis in 6% (29 : 1) acrylamide/bisacrylamide.

### 2.6. Antibodies

Antibodies pRb(IF8)x-sc-102, p107(SD-9)-sc-250, p130(C-20)-sc-317, E2F1(KH-95)-sc-251x, E2F4(C-20)x-sc-866x, (C-108)x-sc-512, E2f5(E19)x-sc-999x, Dp1(K-20)x-sc610x, and Dp2(G12)-sc-849 were purchased from Santa Cruz Biotechnology (USA), pRb(554136) clone G3-245 was purchased from BD Biosciences (USA), and monoclonal anti *β*-actin, clone AC-15 from Sigma (USA).

### 2.7. Statistical Analysis

Growth curves and Western blot experiments were conducted in triplicate, and proliferation rate, cell cycle cytometry, and gel shift assay were performed in duplicate. The values were expressed as mean ± SD using Excel 2010. GelAnalyzer 2010 software was used to quantify the Western blot data. This study used automatic band detection and then background subtraction in the rolling ball mode. The raw volume values of the target protein bands were divided into values of *β*-actin bands on the same lanes. Statistical analysis was performed to compare the mean values between the two groups using Student's test (*p* < 0.05).

## 3. Results

### 3.1. The Growth and Cell Cycle Characteristics of MSCs

**Figure 1:**
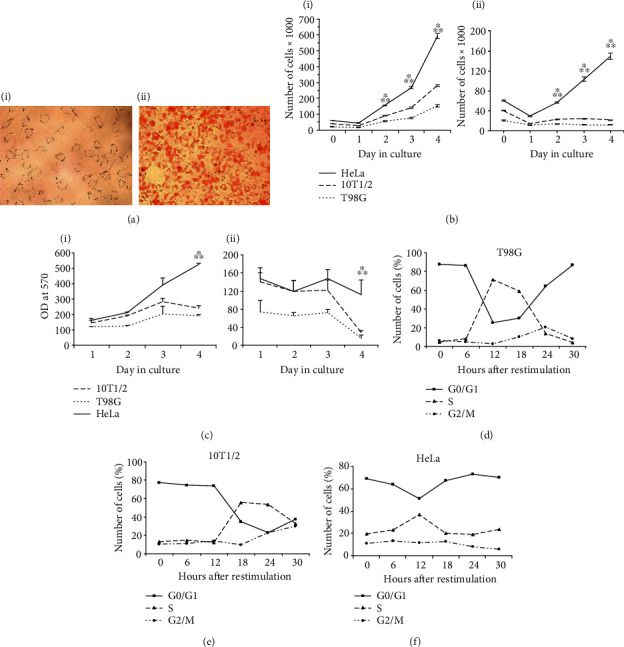
MSCs of the 10T1/2 cell line show serum-dependent growth restriction and cell cycle-dependent oscillation. (a) 10T1/2 cell line possesses the ability to adipocyte differentiation. The cells were observed before AD (*i*) and in 10 days *(ii*) after the induction of AD and Red Oil staining under the Pascal microscope (Carl Zeiss, Germany) with transmitted light using a 10x objective. (b) Growth curves of T98G, 10T1/2, and HeLa cell lines: *i*—full growth medium, *ii*—serum-deprived medium. Equal cell numbers of each line were plated into wells of 96 cell culture plate in triplicate and counted every day until day 4. The data are presented as mean ± SD, ^∗^—*p* < 0.01 compared to the 10T1/2 cells, ^∗∗^—*p* < 0.01 compared to the T98G cells. (c) Cell proliferation rate of the 10T1/2 cells: *i*—full growth medium, *ii*—serum-deprived medium. The cell proliferation rate was estimated using the MTT assay. The asynchronously growing 10T1/2, T98G, and HeLa cells were seeded at a concentration of 2 × 10^4^ cells/well in 100 *μ*l full culture medium in 24 wells of 96-well cell culture plate and incubated for 24 h. Then, 12 wells containing cells of each cell line were filled with full growth medium (FM) while the other 12—with serum-free medium (SFM). For the next four days, the cells were treated with the MTT reagent followed by measuring OD by ELISA reader at 570 nm. The data are presented as mean ± SD, ^∗^—*p* < 0.05 compared to the 10T1/2 cells, ^∗∗^—*p* < 0.05 compared to the T98G cells. (d–f) Flow cytometry analysis of cell cycle progression of T98G, MSCs, and HeLa cell lines, accordingly. The cells were cultured for 72 h in DMEM with 0.15% FCS, restimulated with 10% FCS, and used for flow cytometry in 6 h intervals after restimulation.

The 10T1/2 cell line is composed of multipotent MSCs capable of getting differentiated into adipocytes, myocytes, and chondrocytes. Ten days after the induction of adipogenic differentiation (AD), all the treated 10T1/2 cells accumulated fat vacuoles that were revealed when stained with Oil Red (Figure 1(a), i and ii). In our previous work, the 10T1/2 cells were induced to skeletal muscle differentiation and produced a muscle differentiation marker desmin after treatment with 5-azacytidin. The cells of all the used cell lines grew exponentially until day four in the full growth medium, however, the growth rate of HeLa cells was found to be highest compared to other cell types (Figure 1(b), i). Serum deprivation decreased but did not stop the growth of HeLa cells in contrast to T98G and 10T1/2 cells which stopped dividing (Figure 1(b), ii). The Proliferation rate of the HeLa cells exceeded that of 10T1/2 and T98G cells undergrowth in full or serum-deprived medium (Figure 1(c), i and ii).

The FACS analysis showed that 88% of the T98G cells that were cultured for 72 h under serum-deprived conditions were accumulated in the G0/G1 phase. Furthermore, the 10T1/2 cells were less sensitive to the serum-deprived conditions compared to the T98G cells (Figure 1(d)). Additionally, the G1/S transition of T98G cells was characterized by a sharp increase in the amounts of S phase cells from 8% at 6 h to 72% at 12 h point. At the 30 h point, these cells completed the first cell cycle (Figure 1(d)). The 10T1/2 cells began the G1/S transition later than the T98G cells and the number of S phase cells increased from 12% at 12 h point to 56% at 18 h point (Figure 1(i)). In HeLa cells, the G1/S transition was not as evident as in the above-outlined cell lines, and the number of cells at the S phase were seen to gradually increase from the 6 h point and reach a maximum of 37% at the 12 h point. At 18 h point, the HeLa cells' distribution between the different cell cycle phases was found to be similar to that at the starting point (Figure 1(f)).

### 3.2. Cell Cycle-Dependent Oscillations of p130 and pRb in MSCs

**Figure 2:**
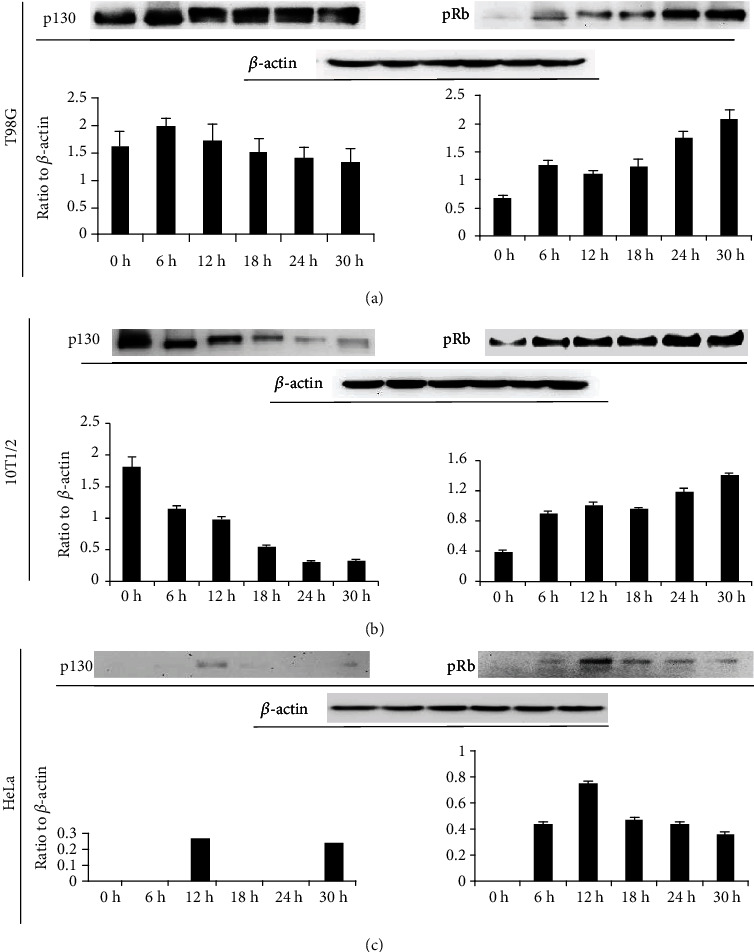
Western blotting analysis of the p130 and pRb cell cycle-dependent oscillations in 10T1/2, T98G, and HeLa cell lines. The cells of all cell lines were synchronized as described above. The cell extracts containing 40 *μ*g of total protein were resolved on 8% SDS–PAGE for the following Western blotting: (a) T98G cell line; (b) MSCs (10 T1/2 cell line); (c) HeLa cell line. The data are presented as mean ± SD.

The T98G cells accumulated at the 0 h and 6 h points fast migrating forms of p130 and pRb (Figure 2(a)). At the 12 h time point corresponding to the G1/S transition, both the proteins began migrating slowly due to phosphorylation [10] (Figure 2(a)). During the next stages of the cell cycle, the steady-state levels of p130 decreased in while that of pRb continuously increased in until the 30 h point. In the 10T1/2 cells, the p130 and pRb levels and mobility at the 0 and 6 h points were extremely similar to those in the T98G cells (Figure 2(b)). Later, the mobility of the p130 increased in until the 18 h point and then decreased in while the steady-state levels of p130 sharply decreased after 12 h point until the end of the observation. The levels of pRb continuously increased its electrophoretic mobility did not change (Figure 2(b)). In HeLa cells, the p130 was observed on a minimal level at the 12 h and 30 h points and not detected otherwise. The pRb levels were found to be low, and this protein exhibited a decrease in electrophoretic motility and an increase in the steady-state levels until the 12 h point. Following this, the levels of pRb slowed down until the end of the observation while its motility did not change (Figure 2(c)).

### 3.3. The Structure of Pp-E2F-DNA Complexes in Quiescent and Cycling T98G Cells

**Figure 3:**
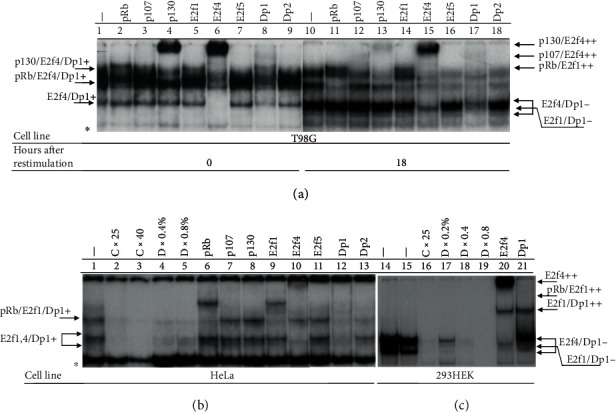
The analysis of the protein structure of the pocket proteins (pp)-E2f-DNA complexes in the T98G, HeLa, and the 293HEK cells by EMSA. (a) The proteins composing the pp-E2f-DNA complexes in the quiescent and cycling T98G cells. (b) The structure of the pp-E2f-DNA complexes in the cycling HeLa cells. (c) The pp-E2f-DNA complexes are completely absent in the 293HEK cells. The samples prepared from the cycling cells were treated with competitor—C, deoxycholate—D, or different antibodies as indicated. The cycling T98G, HeLa, and 293HEK cells were used, accordingly, in the 18 h and 12 h points after restimulation. The protein-DNA bands that included different proteins designated with (+), the band abolishment—(-), the supershifts induced by specific antibodies—(++), and nonspecific bands—(∗).

Two major E2f-DNA bands were observed in the growth arrested T98G cells. The “free” E2f activity showed higher electrophoretic mobility compared to other E2f complexes that included pocket proteins (pp-E2f-DNA) (Figure 3(a)). The free E2fs consisted of only E2f4/Dp1 proteins whose antibody induced corresponding supershifts (Figure 3(a), lines 6 and 8). The slower migrated pp-E2f-DNA activity was presented by one extended band that contains mostly p130-E2f4-Dp1 and a minimal amount of pRb (Figure 3(a), lines 2, 4, 6, 8). In the cycling T98G cells, the amount of free E2f clearly increased compared to quiescent cells due to the elevation of the levels of free E2f4 and appearance of free E2f1. The free E2f4 was presented by two bands while E2f1 migrated as a single band between the E2f4 bands (Figure 3(a), lines 14 and 15). Both E2f4 and E2f1 formed dimers with Dp1 (Figure 3(a), line 17). Additionally, the pp-E2f-DNA activity was divided in the cycling T98G cells into two separate bands (Figure 3(a), line 10). The faster-migrating band consisted of pRb and E2f1 (Figure 3(a), lines 11, 14, and 17). The upper pp-E2f-DNA band in proliferating cells included in addition to p130, E2f4, and Dp1 (Figure 3(a), lines 13, 15, and 17) a little amount of p107 (Figure 3(a), line 12). The levels of p130-E2f4-DNA evidently decreased in cycling compared to that in the quiescent cells (Figure 3(a), lines 13 and 15 and 4 and 6, accordingly).

The HeLa cells constituted the pRb-E2f-DNA and free E2f complexes but did not form the p130/p107-E2f-DNA activities. Similar to the cycling T98G cells, the free E2f complexes in HeLa cells included E2f1-4 (Figure 3(b), lines 9 and 10). These free E2fs were sensitive to specific competitor (C) but retained after deoxycholate (D) treatment (Figure 3(b), lines 2-5). The pp-E2f-DNA complex in the HeLa cells contained pRb-E2f1-Dp1 (Figure 3(b), lines 6, 9, and 12). Furthermore, the treatment with antibody to p107 or p130 did not induce any change in the patterns of the E2f-DNA bands in these cells (Figure 3(b), lines 7 and 8). Notably, the 293HEK cells that originate from the human kidney epithelium transformed with E1A virus oncoprotein did not form any pp-E2f-DNA complexes and contained only free E2f, which consisted mostly of E2f4 (Figure 3(c), lines 14 and 15). The antibody to E2f4 or Dp1 supershifted most of the free E2f activity in this cell line (Figure 3(c), lines 20 and 21).

### 3.4. E2f-DNA Complexes in Quiescent 10T1/2 Cells

**Figure 4:**
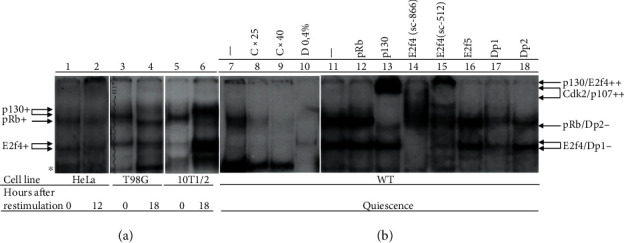
The structure of the pp-E2f-DNA complexes in quiescent 10T1/2 cells. (a) The pp-E2f4-DNA complexes in the quiescent and cycling T98G, 10T1/2, and HeLa cell lines. (b) Analysis of protein structure of the pp-E2f-DNA complexes in the quiescent 10T1/2 cells. The cells were synchronized as described above. The abbreviations are the same as in Figure 3.

The nuclear cell extracts of the quiescent 10T1/2 cells contain several visible E2f-DNA bands, which were specifically abolished by treatment using competitor (C) while deoxycholate (D) was able to abolish slow but not fast migrating E2f-DNA bands (Figure 4(b), lines 8–10). These free E2fs included E2f4 and Dp1 that were supershifted with specific antibodies (Figure 4(b), lines 14, 15, and 17). Furthermore, the 10T1/2 cells, similar to the T98G cells, accumulated in quiescence one pp-E2f4-DNA band, which was divided into p130/p107-E2f4-DNA and pRb-E2f1-DNA bands in cycling cells (Figure 4(a), lines 3-6). The pp-E2f-DNA band in the quiescent 10T1/2 cells included in its uppermost part p130, which was completely supershifted by a specific antibody (Figure 4(a), line 13). Interestingly, E2f4, which plays the role of p130's partner in the T98G cells (Figure 3(a), line 6), was found to be insensitive to the treatment with the same antibody (sc-866) in the 10T1/2 cells (Figure 4(b), line 14) but partly supershifted with the antibody sc-512 that presumably recognized a different compared to the sc-866 antibody epitope in E2f4 (Figure 4(b), line 15). The pRb containing band was partly supershifted by an antibody against pRb (Figure 4(b), line 12).

## 4. Discussion

MSCs represent a type of multipotent somatic stem cells for nonhematopoietic tissues capable of being differentiated into bone, fat, and other lineages of connective tissue [23, 24]. They make the fate choice similar to other types of stem cell cells in the course of division coupled with the loss of multipotentiality. It is believed that commitment to differentiation occurs at the early G1 phase that lasts until the G1 restriction point and is under the control of pRb proteins [1, 25]. Additionally, self-renewing ESCs have a very short G1 phase and are not induced to differentiation in the course of continuous proliferation. Furthermore, pRb signaling is not active in self-renewing ESCs but activates in differentiating ESCs. This allows pocket proteins to regulate the expression of the OCT4 and other genes associated with pluripotency [26, 27]. MSCs derived from different tissues similar to ESCs are characterized by active proliferation in vitro. In contrast to ESCs, MSCs' cell cycle parameters can be compared with those in somatic differentiated cells [28]. The mechanisms of the cell cycle regulating the exiting and reentering and maintenance of transient quiescence are ubiquitously based on pRb-E2f signaling, however, specific roles for separate pRb family members in MSCs' cell cycle have not been studied. Publications show that acute pRb loss results in cell cycle reentry in mouse embryonic fibroblasts and stem cells from various tissues [3, 29]. However, RB-/- single and 130-/-; p107-/- double knockout mice undergo cell cycle arrest that may be abolished by triple knockout for the RB-related genes [2]. Other evidences have pointed out that the regulation of transient and long-term quiescence is linked to the accumulation of p130, which suppresses the activity of genes associated with S and G2/M phases [30, 31]. In *C. elegans*, *Drosophila*, and mammals, the DREAM complex was identified which includes Dp, E2f4, and p130/p107 but not pRb as the core components. DREAM regulates quiescence via the suppression of more than 800 genes associated with cell cycle progression [32, 33]. In addition to single E2F site, DREAM can bind DNA through single CHR (cell cycle homology region) or combined CDE (cell cycle-dependent element) and CLE (CHR-like element) [34]. The formation of DREAM is mediated by the activation of the p53 tumor suppressor which transmits the suppressive signal through p53-p21-DREAM-E2F/CHR (p53-DREAM) pathway [35]. The human papilloma virus E7 protein has been shown to bind to the p130 and compromise the tumor suppressive function of p53 [36]. In summary, both p130 and pRb regulate the G1 checkpoint through the E2f site; however, the p130 pathway is different in comparison to pRb signaling, it accumulates more signals and makes the key contribution to this regulation.

This study found that under serum-deprived conditions, MSCs, similar to the T98G cells, enter quiescence in contrast to HeLa cells that continued to proliferate. Both MSCs and T98G cells were seen to produce p130 cell cycle regulator, which showed the cell cycle-dependent oscillations associated with its phosphorylation. Additionally, the p130 was presented in its active hypophosphorylated form in the quiescent cells and was hyperphosphorylated and ubiquitylated at G1/S transition, which resulted in its decrease during S and G2/M phases in the T98G cells [10, 20, 22] and disappearance in MSCs (Figure 2(b)). In contrast to p130, the steady-state level of pRb was continuously elevated due to its phosphorylation during the cell cycle in both cell lines [10]. No constitutive expression of p130 was observed in HeLa cells while pRb was produced and showed maximal level at the 12 h point corresponding to the G1/S transition (Figure 2(c)). Furthermore, it was found to form a complex with E2f4/Dp1 in the quiescent T98G cells and MSCs, which was detected by specific antibodies in EMSA. Upon reentering the cell cycle, this complex in the T98G and the 10T1/2 cells was exchanged for the formation of the pRb-E2f1-DNA complex. The p130-E2f4-DNA complex was absent in the HeLa cells, although these cells produced pRb and formed the pRb-E2f1-DNA complex. To this end, the p130 was found to be inactivated in the 293HEK cells by E1A of adenovirus [37]. Notably, both pRb and p130 were produced in MSCs on the levels similar to that in the T98G cells with functional pocket proteins. pRb showed low sensitivity in EMSA to the treatment with a specific antibody similar to E2f4. Possibly, conformational epitopes that formed pRb and E2f4 bound to DNA in MSCs are different compared to that in the T98G cells.

In summary, the results of this study support the idea that the major effector protein regulating entering, exiting, and the maintenance of quiescence in MSCs, similar to the T98G cells with functional pocket proteins, is the p130 and not pRb. This function of p130 is conserved in development and mediated by its ability to form stable p130-E2f4-DNA complexes that repress the genes required for G1/S transition, replication, and mitosis.

## Data Availability

The underlying data are available under request.

## Abbreviations

FCS: Fetal calf serum
EMSA: Electrophoretic mobility gel shift assay
pRb: The retinoblastoma susceptibility gene product
ESCs: Embryonic stem cells
MSCs: Mesenchymal stem cells.
